# Genomic Effects of the Vitamin D Receptor: Potentially the Link between Vitamin D, Immune Cells, and Multiple Sclerosis

**DOI:** 10.3389/fimmu.2018.00477

**Published:** 2018-03-12

**Authors:** Ming Lu, Bruce V. Taylor, Heinrich Körner

**Affiliations:** ^1^Menzies Institute for Medical Research Tasmania, Hobart, TAS, Australia; ^2^Institute of Clinical Pharmacology, Anhui Medical University, Key Laboratory of Anti-inflammatory and Immunopharmacology, Ministry of Education, Engineering Technology Research Center of Anti-inflammatory and Immunodrugs in Anhui Province, Hefei, China

**Keywords:** multiple sclerosis, vitamin D, immune system, environment, genetics

## Abstract

Vitamin D has a plethora of functions that are important for the maintenance of general health and in particular, the functional integrity of the immune system, such as promoting an anti-inflammatory cytokine profile and reducing the Treg/Th17 ratio. Multiple sclerosis (MS) is a chronic, inflammatory, and neurodegenerative central nervous system (CNS) disorder of probable autoimmune origin. MS is characterized by recurring or progressive demyelination and degeneration of the CNS due in part to a misguided immune response to as yet undefined (CNS) antigens, potentially including myelin basic protein and proteolipid protein. MS has also been shown to be associated significantly with environmental factors such as the lack of vitamin D. The role of vitamin D in the pathogenesis and progression of MS is complex. Recent genetic studies have shown that various common MS-associated risk-single-nucleotide polymorphisms (SNPs) are located within or in the vicinity of genes associated with the complex metabolism of vitamin D. The functional aspects of these genetic associations may be explained either by a direct SNP-associated loss- or gain-of-function in a vitamin D-associated gene or due to a change in the regulation of gene expression in certain immune cell types. The development of new genetic tools using next-generation sequencing: e.g., chromatin immunoprecipitation sequencing (ChIP-seq) and the accompanying rapid progress of epigenomics has made it possible to recognize that the association between vitamin D and MS could be based on the extensive and characteristic genomic binding of the vitamin D receptor (VDR). Therefore, it is important to analyze comprehensively the spatiotemporal VDR binding patterns that have been identified using ChIP-seq in multiple immune cell types to reveal an integral profile of genomic VDR interaction. In summary, the aim of this review is to connect genomic effects vitamin D has on immune cells with MS and thus, to contribute to a better understanding of the influence of vitamin D on the etiology and the pathogenesis of this complex autoimmune disease.

## Introduction

Vitamin D was named as the fourth factor identified in an experimental rickets model (1). The role of sunlight in the synthesis of this factor had been recognized in unrelated research but had initially not been understood in the context of this factor now termed vitamin D (2). Further research on the constitution of sterols and their connection to vitamins resulted in the Nobel price for Chemistry in 1928 for Adolf Windaus (3). Finally, the biochemical pathway in its entirety and the connection to sunlight was published in 1955 (4) and summarized in more detail 25 years later (5).

The nomenclature of vitamin D and its metabolites has been well defined by Vieth, and for the purpose of this review we will use their terminology to refer to the level of serum 25 hydroxy vitamin D [25(OH)D] the major circulating form of vitamin D that is often used to define vitamin D status (6). For *in vitro* experiments, we use 25(OH)D_3_ and 1,25(OH)_2_D_3_ according to the quoted work.

As an important environmental factor, vitamin D deficiency has been associated with increased multiple sclerosis (MS) risk (7, 8), a finding that has been supported genetically by a Mendelian Randomization analysis of vitamin D-associated single-nucleotide polymorphisms (SNPs) (9, 10). Moreover, multiple factors that affect vitamin D status including ultraviolet B radiation exposure (UVR), latitude, systemic infection, and smoking, are associated with MS risk, and higher levels of serum 25(OH)D have a protective effect on MS risk but not on the clinical course or the severity of MS (11). Although clinical trials of vitamin D supplementation with the primary outcome being MS risk have not been undertaken due to their complexity, the need for many years of follow-up and the overall low risk of MS in the general population there is a considerable body of research regarding the protective effect of vitamin D status on MS clinical activity, such as a decrease in magnetic resonance imaging lesions (12) and a reduced hazard of relapse (13). Since MS is a chronic, inflammatory, autoimmune disease that could potentially originate from an autoimmune response to neurodegenerative central nervous system (CNS) antigens such as protein components of the myelin sheath (14) with periods of de- and remyelination or progressive demyelination driven by a strong involvement of various branches of the immune system (15) an effect of vitamin D metabolites on the overall inflammatory state within the CNS would be a logical explanation (11).

Furthermore, besides the obvious impact of a deficiency due to environmental factors, genetic aspects of control over the vitamin D metabolism appear to be also important. In total, more than 200 common risk SNPs have been found in genome-wide association studies (GWAS) outside the HLA region that are significantly associated with MS (16). Among these common risk SNPs [published in the NHGRI GWAS Catalog (https://www.ebi.ac.uk/gwas/search?query=MULTIPLE%20SCLEROSIS) and IMSGS (16)], there are several that are linked with vitamin D metabolism-associated genes. CYP24A1, rs2248137 (16), and rs2248359 (17), and SNPs that tag a chromosome 12 linkage disequilibrium (LD) block that contains the gene CYP27B1, rs12368653/rs703842/rs10876994 (17, 18), rs201202118 (19), and rs701006 (16). Some rare risk SNPs have been identified in vitamin D metabolism genes, such as rs118204009 in CYP27B1 (20) although this has not been confirmed in subsequent work (21), and rs117913124 in CYP2R1 (22), which may impact both the risk of vitamin D insufficiency and the risk to develop MS, a validation of these findings in other cohorts will be necessary. However, the cross sectional case–control design of GWAS that define risk is by their nature not suitable for predicting clinical course. All known genes identified by GWAS including those identified in the HLA region have not shown a significant association with the clinical course of MS in the GWAS studies themselves. Some associations with MS clinical course have been found when these SNPs are used as candidate genes in longitudinal MS studies focusing on clinical severity and disease course (23–26) suggesting that genetic variants that tag the Vit D pathway genes have a functional impact on the clinical course of MS (27, 28).

A second major aspect of the role of vitamin D in the etiology of MS are the extensive genomic binding regions of the nuclear vitamin D receptor (VDR), which is the only cognate receptor of the active form of vitamin D [1,25(OH)_2_D_3_ or calcitriol]. It acts as a transcription factor (TF) that interacts with multiple other TFs and coregulators, and binds to regulatory hotspots throughout the genome (29, 30). In fact, many enhancers that contain VDR-binding sites are located within regulatory hubs of enhancers [so-called super-enhancers (SEs)] which are now considered to be involved in the regulation of cell fate (cell identity or cell differentiation) (29, 31). The large number of genes containing VDR-binding sites is probably one mechanism that allows organisms to integrate environmental stimuli into functional effects which can influence for example, cell differentiation (30, 32–34).

In this review, we try to connect the genomic effects of vitamin D on immune cells with MS etiology by discussing the cistrome of the VDR. First, we will discuss the effects of vitamin D on immune cells and the implications of GWAS results that indicate vitamin D-associated genes are involved in the immune response. Furthermore, we will review the cistrome of the VDR in multiple immune cells. The association of the VDR cistrome with epigenomic characteristics, including SE regions, in these cells, could help to analyze the potential mechanisms underlying the role of vitamin D in MS etiology and pathogenesis.

## The Influence of Vitamin D and its Complex Metabolism on MS

### Immunological Effects Underlying the Role of Vitamin D As an Environmental Factor for MS

The early stages of MS are assumed to be mediated by activated CD4^+^ T cells that express pro-inflammatory cytokines and facilitate chronic inflammation that causes demyelination. Originally, the concept of an ill-timed transition from the Th1 to Th2 state was considered to be a major contributing factor for the development of autoimmune disease (35). However, new, more detailed studies that have investigated the roles of newly defined T cell subsets under the extended T cell paradigm point to an imbalance of Th17 and Treg populations as an underlying factor for the development of multiple autoimmune diseases (36–39).

The active form 1,25(OH)_2_D_3_ has been implicated previously in Th1/Th2 conversion and now in Th17/Treg balance (40, 41). Specifically, 1,25(OH)_2_D_3_ can inhibit the Th17 phenotype by inhibiting the transcription of *ROR*γ*t, IL-17, IL-23R*, and *IL-22* (42), and promote the Treg subset by inducing the expression of *IL-10, Foxp3*, and *CTLA-4* (43, 44). Furthermore, 1,25(OH)_2_D_3_ can also inhibit the expression of *IL-2* and *IFN-*γ and modulate differentiation of Th17 cells (45, 46). Granulocyte/macrophage colony-stimulating factor (GM-CSF) secretion, as an emerging risk factor for MS, is also inhibited by 1,25(OH)_2_D_3_ (36, 47). In addition, the expression of the Th17 marker molecule CCR6 is reduced by 1,25(OH)_2_D_3_, which in turn, reduces the number of Th17 cells migrating in response to CCL20 to the central nervous system (48). Furthermore, 1,25(OH)_2_D_3_ also inhibits the proliferation of isolated CD4^+^ T cells and myelin basic protein (MBP)-specific T cells from MS patients *in vitro* (49).

In addition to changes in the Th subset ratio, other immune cell subsets also potentially play roles in MS pathology, which can be aligned with or complement T cell-centered pathology. Pro-inflammatory T cells can recruit B cells, myeloid cells [monocytes/dendritic cells (DCs)], natural killer (NK) cells, or CNS-resident macrophages (microglia) to the pathology. They can interact and, for example, induce complement depositions and cause opsonization and local activation of microglia and macrophages until demyelination occurs (50). As immunoglobulin G1 and myelin reactive antibodies are identified in the cerebrospinal fluid of MS patients, B cells may contribute to MS pathogenesis *via* their ability of antigen presentation or production of immunoglobulins (50). Pro-inflammatory CD14^+^CD16^+^ monocytes show activation in MS patients, and their ability to disrupt the blood–brain barrier also is contributing to MS pathology. Monocyte differentiation can lead to opposite functionality in macrophages depending on the cytokine environment: either pro-inflammatory M1- (or DC1) or anti-inflammatory M2-macrophages (or tolerogenic DC2) (51, 52). NK cells can migrate into CNS and can display various functions. They can show cytotoxicity to DCs suggesting a pathological role in MS. Counterintuitively, their number and functional activity is reduced in MS patients (50).

By analysis of gene expression patterns (such as RT-PCR and RNA-seq) or epigenomic features [such as chromatin immunoprecipitation sequencing (ChIP-seq) and DNase hypersensitivity sites], all immune cells (including microglia) and neuron cells partly express MS risk genes (16, 53, 54), but MS risk SNPs are preferentially more enriched in the SE region of CD4^+^ T cells, than in that of B cells and monocytes (32, 33). Interestingly, ChIP-seq results showed a stronger sensitivity of MS risk genes detecting 121 distinct genes with VDR binding peaks in DC2s whereas RNA-seq identified only one differently expressed gene in DC2s compared with the transcriptome of DC1s or monocytes (53). Supporting the notion that vitamin D has a biological role in CNS autoimmunity, all these cell types can be impacted by 1,25(OH)_2_D_3_. For example, 1,25(OH)_2_D_3_ inhibits proliferation, plasma cell differentiation and antibody production of B cells, but promotes class-switched memory B cell generation and B cell apoptosis (55). Furthermore, *via* inhibiting cell differentiation 1,25(OH)_2_D_3_ could be involved in preserving the immature status of DC with considerable ramifications for the specific immune response. This cell population has in its immature form characteristics such as lower expression of MHC class II, costimulatory molecules and IL-2, which could lead to a modulation of antigen presentation and ultimately, self-tolerance (55).

In the context of these vitamin D functions the still unresolved question of the required, physiologically effective concentration of 1,25(OH)_2_D_3_ should be mentioned as a caveat. The concentrations employed in *in vitro* experiments (10–100 nM) or the physiologically effective concentrations observed in the *in vivo* microenvironment (1–10 nM) can actually be produced in both autocrine or paracrine pathways by DCs or T cells *via* the hydroxylation of 25(OH)D (which has on average a physiological concentration in the blood of 50–80 nM), to 1,25(OH)_2_D_3_ by the enzyme 25-hydroxyvitamin D_3_ 1α-hydroxylase (CYP27B1) (43, 46, 56). Notably, concentrations of either 10^−6^ or 10^−7^ M of 1,25(OH)_2_D_3_ will induce either *Foxp3* or *IL-10* expression, respectively, with little co-expression (44). This suggests that a precise control of the local vitamin D concentration needs to be in place in the microenvironment to achieve homeostasis. How this is approached physiologically is not understood. Actually, under *in vivo* conditions a small rise (~10 nM) in 25(OH)D_3_ serum levels can lead to significant changes at hundreds of sites within the epigenome of human leukocytes (57).

The complexity of the interactions between vitamin D and the immune system have been demonstrated in more detail in mouse models. In experimental autoimmune encephalomyelitis (EAE), a model of neuroinflammation used to study MS, vitamin D treatment ameliorated the clinical symptoms of EAE mice. Reciprocal bone marrow chimeras using VDR-knockout (VDR-KO) bone marrow showed that the hematopoietic donor cells needed to have a functional VDR to cause this attenuation of the pathology. Interestingly, no change in the total number of CD4^+^ T cells or the proportion of Foxp3^+^ T-cells in the periphery or the CNS could be detected (58). By contrast, in a model of inflammatory bowel disease either in VDR-KO mice or in the absence of vitamin D an overproduction of Th17 cells and a reduction of inducible Tregs could be shown (59). Addition of vitamin D reversed this effect in T cell culture (59) and in an *in vivo* model (60). In a dextran sodium sulfate colitis model, VDR-KO mice showed increased sensitivity and early mortality which could be ameliorated with 1,25(OH)_2_D_3_ (61). However, ultimately a T cell-specific VDR-KO model will have to be employed to confirm these observations. Furthermore, VDR-KO mice have an impaired resistance to allergic asthma, supporting the notion of a suppressive effect of VDR absence on Th2 responses (62). Controversially, it has also been described that less Th2-cytokines IL-4, -5, and -13 are produced by VDR-KO Th2 cells (63), and vitamin D can inhibit the allergic asthma phenotype (64). Furthermore, gene conservation between primates and rodents needs to be considered. For example, VDR target genes encoding AMPs human beta-defensin 2 and cathelicidin (CAMP) and the pattern recognition receptor NOD2 are only induced in human cells after 1,25(OH)_2_D treatment, but not in mouse cells (65).

The immunoregulatory effects of vitamin D on the human immune system will be more complex, as a result of synergistic/antagonistic effects of various environmental factors with vitamin D, and dose-/time-dependency *in vivo*. A multitude of clinical translational studies, in part double-blinded randomized controlled trials on clinically isolated syndrome or relapse-remitting multiple sclerosis (RRMS) MS patients, has attempted to analyze the role of vitamin D. It has been a consistent result that serum 25(OH)D level and TGF-β level will increase after vitamin D supplement of either 1,000 IU/day for 6 months (66), or 20,000 IU/week for 48 weeks (supplementing IFNβ-1b therapy) (67), although one study (7,000 IU/day for 4 weeks followed by 14,000 IU/day for 44 weeks in addition to IFNβ-1a therapy) only observed an increased serum TGF-β level in the placebo group rather than the vitamin D group (68). However, most studies have not been able to reveal an immunoregulatory effect of vitamin D on serum cytokine profile and immune cell subsets (66, 67, 69–72). This could indicate that there is no correlation between the higher serum 25(OH)D level and pro-inflammatory cytokine/chemokine profiles (39). One study observed a reduced proportion of CD25^+^ Tregs and decreased circulating soluble-CD25 only in placebo group, suggesting a maintenance effect of vitamin D on immune homeostasis (73). Other studies also found a reduced IL4^+^ Th cell proportion in the placebo group (68), and the IFN-γ/IL-4 ratio (Th1/Th2-balance) was more directed toward IL-4 in patients with increased serum 25(OH)D levels (74). This would suggest an anti-inflammatory effect of vitamin D. Among those studies, a proposed dose-dependency is reflected in high-dose 10,400 IU/day vitamin D treatment for 6 months will increase 25(OH)D level more strongly and can alter the proportion of CD4^+^ T cell subsets (i.e., reduced IL-17^+^ Th/CD161^+^ Th/effector memory Th cells, increased central memory Th/naive Th cells) in RRMS patient is compared with 800 IU/day (75). Similarly, 800 IU/day vitamin D treatment for 1 year will induce more serum IL-17 in RRMS patients, while patients with high-dose 4,370 IU/day treatment only showed heterogeneous IL-17 response (70). Concurrently, a time-dependency of vitamin D treatment is demonstrated by reduced anti-EBNA1 protein and fragment antibody levels from baseline to week 48 but not to week 96 of the therapy (76).

Interestingly, some MS-specific changes that can not be observed directly *ex vivo* can be found after PBMCs culture *in vitro*. For example, in tissue culture the GM-CSF secretion of CD4^+^ T cells from MS patients was less reduced by vitamin D than in healthy controls (77). In addition, the serum 25(OH)D levels from RRMS patients correlated positively with the ability of Treg cells to inhibit T cell proliferation (74). Likewise, for PBMCs from MS patients with vitamin D supplement, their *in vitro* proliferative responses to antigens such as MBP were reduced (69). Similarly, *in vitro* PBMCs proliferation in the vitamin D supplement MS group were significantly reduced, and the supernatant levels of TGF-β and IL-10 were increased, compared with that in the placebo MS group (78). In consideration of the complexity of vitamin D supplement effects *in vivo*, more RCT trials with higher statistical power have to be carried out to avoid heterogeneity among participants and different centers. This can be supplemented by Real-World data, such as patient-reported outcomes or associated epidemiology data (79). On the other hand, analyses of genomic effects of vitamin D and its associated genes in different cell types could provide a more reductionistic explanation of confusing results from translational researches.

### Potential Mechanism of Vitamin D-Associated Metabolic Enzymes in MS

The immune cell types discussed earlier such as T cells (49, 56, 80–83), B cells (84, 85), DCs (86–89), macrophages (87), and CNS cells, such as neurons, microglias, astrocytes, and invading lymphocytes (90–92), can express *CYP27B1* and *CYP24A1*, according to their environmental status and during different cell stages. Interestingly, these genes are under precise regulation as a result of integrating environmental factors and interactions among different cell types. For example, after 24 h activation of T cells, the 1,25(OH)_2_D_3_ production from 25(OH)D_3_ is very low, but it strongly increases after 48 h of activation (82). Therefore, after only 24 h activation, some investigations cannot observe the capacity of CD4^+^ T cells for converting 25(OH)D_3_ to 1,25(OH)_2_D_3_ (56). Under normal physiological conditions, inactivated T cells lack 1α-hydroxylase protein, and the function of the CYP27B1 enzyme will be provided by APCs in close contact (52, 56). Furthermore, activated T cells that interact with macrophages will increase the *CYP27B1* expression in macrophages, an effect that is similar to the treatment of macrophages with IFN-γ or soluble CD40L. In fact, multiple environmental factors and cell signals impact the regulatory network of *CYP27B1 and CYP24A1* and change their expression level, such as 1,25(OH)_2_D_3_/VDR itself, parathyroid hormone (PTH)/prostaglandin/PKA, ${\text{Ca}}_{2}{\;}^{+}\text{/}PKC$, TCR/Zap-70, and various other cell differentiation signals (41, 56, 80, 82, 83, 88, 93). For example, the binding of STAT1 to VDR which is induced by IFN-γ prevents the *CYP24A1* expression in monocytes and macrophages (94). But Th cell-produced IFN-γ can promote the TLR2/1 induced expression of *CYP27B1* and *VDR* in monocytes, while IL-4 has been shown to be inhibitory and instead induces *CYP24A1* expression (95). Furthermore, through binding to VDR, 1,25(OH)_2_D_3_ can downregulate its own production by decreasing the expression of *CYP27B1* and increasing the expression of *CYP24A1* (96–98). In addition, VDR can also regulate its own expression, and thus, the metabolism of vitamin D *via* binding vitamin D responsive elements in the enhancer/promoter regions of *VDR/CYP24A1/CYP27B1* genes (99, 100). During the process, epigenetic mechanisms participate in the regulation of *VDR* expression, such as methylation of *VDR* promoter, acetylation of histone 4 in *VDR* enhancers, and levels of miR-125b (101). All these studies have revealed a complex regulatory network of *CYP27B1/CYP24A1* with different requirements in different cell types, although, interestingly, *in vitro* myeloid cells express higher levels of *CYP27B1/CYP24A1* without further stimulation (52, 53).

It has been attempted to isolate the potential functions of MS risk SNPs by identifying MS risk genes (with or in proximity to MS risk SNPs) that are associated with heritable altered ratios or responsiveness of immune cells subsets from MS patients (54). Many genes that are stably expressed in certain immune cell subsets and have been identified as potential MS risk genes from cohort studies, direct immune cell differentiation in MS patients such as *NF-κB* in PBMCs (102), *IL2RA* in GM-CSF^+^ memory Th cells (103), *TYK2* (tyrosine kinase 2) in Th2 cells (104), *EOMES/TBX21* in CD56^+^ NK cells (105), and *ZMIZ1* in plasmacytoid DCs (106). However, the mechanisms how those genes with risk alleles affect the responsiveness or differentiation of immune cell subsets are still unclear. For example, questions how *IL2RA* with risk allele alters IL-2 responsiveness of naive Th cells and deviates their differentiation into GM-CSF-producing memory Th cells. How *IL2RA* polymorphism impacts IL-2/STAT5 signaling pathway? *IL2RA* gene actually has a high ranking SE region in all Th1/Th2/Th17 cells, with potentially extensive regulatory function (33). How *TYK2* polymorphism regulates T lymphocyte differentiation toward a Th2 phenotype and what the functions of *EOMES/TBX21* gene sets in CD56^+^ cell and *ZIMZ1* sets in plasmacytoid DCs of MS patients are. Could there be a robust unified underlying mechanism that affects a common key genomic structure involved in cell fate determination? We will discuss the influence of the vitamin D status in genomic regions as a key to these questions in the second part of this review.

In support of a role of vitamin D, MS-associated SNPs that have been identified in recent GWAS results are located around the vitamin D metabolism-associated genes *CYP27B1* and *CYP24A1*, and they have a modest but significant regulatory effect on gene expression in certain cell types.

The gene *CYP27B1* encodes 25-hydroxyvitamin D_3_ 1-alpha-hydroxylase, an enzyme that converts 25(OH)D by hydroxylation to 1,25(OH)_2_D_3_. This hydroxylase forms part of an LD group on chromosome 12 and the major alleles of SNPs rs10877012 (G), rs10877013 (C), and rs703842 (A) (tagged by SNP rs6581155 with *r*^2^ ≥ 0.8) identify the *CYP27B1* haplotype associated with an increased risk of MS (52, 107). It has been shown *in vitro* that this haplotype of *CYP27B1* is downregulated in tolerizing DC which could point to a role in an autoimmune pathology (52). And lower CYP27B1 mRNA expression was associated with rs10877013 (C) in LPS + IFNγ-treated monocytes (108). Unfortunately, another member of this LD *METT21B* (previously termed *FAM119B*) has been shown to be similarly regulated in the same cell types no matter its gene expression from qPCR or its multiple isoforms from RNA-seq, which prevents a clear conclusion (52). In an unrelated study *METT21B* mRNA has been shown to be downregulated in cells isolated from whole blood samples (109). However, according to mRNA data from immortalized peripheral lymphocytes, the expression of *METTL1, CYP27B1*, and *CDK4* are all regulated by rs10876994, rs12368653, or rs703842 (110). Using a comprehensive approach [expression quantitative trait loci (eQTL), ENCODE annotation, and luciferase reporter assay], it has been indicated that the SNP rs10877013 can regulate the enhancer activity and the gene expression of the LD region (increasing the expression of *TSFM/TSPAN31*, reducing the expression of *CYP27B1/METT21B/AVIL*) in a allele-dependent and orientation-dependent way, by interfering with CCAAT/enhancer-binding protein (C/EBP) α and β binding and engaging promoter–enhancer/promoter–promoter interaction loops (107). Interestingly, C/EBP β can determine adipocyte differentiation at early stage by interacting with other TFs (including VDR) in a TF binding hotspot on chromatin (30). And there are multiple isoforms for *METT21B* and *TSFM* in DC2s, suggesting a potential association of the region with long non-coding RNA transcription (111) and further SE regions (112). But in the immune system, the relationship between haplotype and cell fate determination needs further analysis. A second risk SNP (rs2248359 located separately from the chromosome 12 LD) that has been identified in the same GWAS as MS-associated tags the gene *CYP24A1* that encodes for the enzyme 1,25-dihydroxyvitamin D3 24-hydroxylase which can deactivate 1,25(OH)_2_D_3_ (17). The MS risk allele rs2248359-C increases *CYP24A1* expression in the frontal cortex but not in white matter of the human brain (92). This enzyme was also present in DC and 1,25(OH)_2_D_3_-simulated B cells but without a difference between two alleles in tolerizing/pro-inflammatory DC and B cell (52, 108). Taken together, the specific mechanism of how these risk SNPs function in different cell types and contribute to the regulation of gene expression and then the etiology of MS is still unclear as are their possible multiple indirect effects on distant genes or long-term effects amplified by other risk factors during cell activation. It is conceivable that one SNP can impact multiple transcription processes in certain cell type at certain developmental stage within some basic important chromatin structures, such as SE.

## Regulatory Mechanism of VDR in MS

### VDR Binding to Chromatin and Its Association with MS

There are only a limited number of studies about the general effects of VDR binding to VDR binding sites in the genome on the shape of the transcriptome. VDR belongs to the superfamily of nuclear receptors and is located mainly in the cytoplasm. After binding its cognate ligand 1,25(OH)_2_D_3_ VDR recruits its partner retinoid X receptor (RXR) and can now translocate from the cytoplasm to the nucleus and engage target genes *via* binding to its genomic binding sites (113–115). It interacts with TF and/or coregulators. And it binds to cell type-specific VDR binding sites. The association between extensive VDR binding and MS is supported by the overlapping localization of VDR ChIP-seq intervals and MS risk SNP regions (53, 116–118).

#### The Interaction between VDR and TFs/Coregulators

In *in silico* motif analysis of experimentally determined VDR binding sites by ChIP-seq identified that the majority of VDR binding sites did not have canonical VDR binding motifs, which consist of a direct repeat of two hexameric core binding motifs with a spacing by 3 nucleotides (DR3), even though they are enriched for DR3 motifs compared with the genomic background (53, 119, 120). Therefore, to allow DNA binding and the regulation of transcription directly or indirectly, it is mandatory that VDR can form complexes in association with other TFs or coregulators. First, VDR can modify its DNA location by interacting initially with other TFs. Examples are the TF PU.1 (for monocyte/macrophage and B cell differentiation), STAT5 (for cytokine signaling in T cell activation), DNA-binding subunit of GABP (GABPA) in THP-1 cells, with motifs that have been found within the peaks of VDR/RXR binding (119–121). The co-localization of GABPA and VDR has been confirmed by ChIP-seq data of GABPA cistrome, which can further interact with PU.1 for regulating genes in cellular and immune signaling processes (122). Six MS risk genes expressed by myeloid cells (monocyte/DC1/DC2) have also been found co-localized with VDR (both with VDR binding region and canonical VDR motif) (53). One motif of the MS risk gene *BATF* is highly represented in VDR binding peaks of DC1s and DC2s (53), suggesting a potential association between DC and Th17, and a role of VDR in their differentiation (123). Furthermore, sixteen DC1-specific MS risk TF genes, which are only represented in VDR binding peaks of DC1s but not of DC2s/monocytes, are enriched for IL-1 (*p* = 2.6 × 10^−6^)/IL-6 (*p* = 6.1 × 10^−9^)/MIF (*p* = 9.2 × 10^−5^) (not corrected for multiple testing) signaling pathways associated with inflammation (53). By analyzing ChIP-seq data both from VDR and PU.1, PU.1-VDR cross talk has been demonstrated in open chromatin in 1,25(OH)_2_D_3_-sensitive PU.1 loci which were closer to the 1,25(OH)_2_D_3_ target genes (124). It could also be shown by integrating VDR ChIP-seq data and GWAS results that only 2 of 42 identified trait/disease risk SNPs resided within a canonical VDR-binding site, and 33% of the 42 SNPs impacted the immune-related binding and gene regulation by other TFs including NF-κB. The co-enrichment of VDR and NF-κB binding in CEPH cell lines suggested the cross talk between VDR and NF-κB (118). In human CD4^+^ cells, VDR binding intervals are enriched for CCCTC-binding factor (CTCF) motifs (125), which is an architectural protein at the borders of topologically associated domains (TAD). In monocyte, 1,25(OH)_2_D_3_ -dependent CTCF binding sites also partly overlap with VDR binding regions and contribute to DNA looping/TAD shaping (126). VDR/RXR and TF 4/β-catenin can also interact and bind one distal enhancer region to regulate *c-FOS* and *c-MYC* gene expression in colonic cells, which occurs in a ligand-dependent manner (127).

Second, VDR has also been shown to bind VDR coregulators, which do not directly interact with DNA but are associated with the regulation of gene expression by their modulation of the DNA superstructure such as helicase, histone acetyltransferase, proteasome-dependent proteolysis, histone deacetylase, etc. (113). For example, VDR can recruit histone acetyltransferase to activate transcription (128) and bind nuclear receptor co-repressor 1/2 to recruit histone deacetylase which suppresses transcription *via* chromatin modification (113). And in LS180 (human colon adenocarcinoma) cells, VDR co-localize with many other coregulators for regulating regulated 1,25(OH)_2_D_3_ responsive genes (129).

#### Engaging VDR Binding Region and Enhancers

The main DNA regions bound by VDR are enhancers indicating that this nuclear receptor acts with a strong tissue specificity. The observation of autoregulation of VDR on the *VDR* gene, has supported the notion that VDR/RXR can bind the S1, S3 and U1 enhancers in the *VDR* gene regulating its own transcription in a tissue-specific manner (99, 130). The regulation of *VDR* depends on epigenetic modification (101). For example, hypermethylation of VDR promoter and deacetylation of histone H4 in VDR enhancers will reduce the transcription of VDR gene. High levels of miR-125b posttranscriptionally downregulate VDR mRNA levels. Furthermore, coregulatory factors such as RNA polymerase II, cAMP response element-binding factor (CREB), glucocorticoid receptor, C/EBPβ, and Runt-related transcription factor 2 can be recruited to regulate *VDR* expression after ligand-dependent epigenetic modification, such as histone H4 acetylation (99). This suggests that these *VDR*-specific enhancers can integrate signals from multiple environmental factors (e.g., vitamin D, glucocorticoids, retinoid acid, and PTH) by interacting with their associated TFs (99, 130). This is also the case for a central metabolic enzyme of the vitamin D pathway, 1,25(OH)_2_D_3_ 24-hydroxylase or CYP24A1. In the case of this enzyme, the presence of 1,25(OH)_2_D_3_ strongly induces the simultaneous presence of VDR and RXR. After translocation to the nucleus the VDR complex binds to VDR binding sites at the promoter regions of *CYP24A1* and to a cluster of enhancers located in intergenic regions downstream of the enzyme upregulating its expression. In this case, the recruitment of coregulators steroid receptor coactivator 1, mediator complex subunit 1 (MED1), silencing mediator of retinoid and thyroid hormone receptor, and the occurrence of histone H4 acetylation are all associated with the VDR/RXR binding (17, 131, 132).

With the development of ChIP-seq, which represents an unbiased method that can quantify binding of a specific protein to certain DNA sequences, it is possible to determine the genome-wide extent of the binding of a specific TF to DNA. In a B cell-derived lymphoblastoid cell line (LCL), 1,25(OH)_2_D_3_-treated samples have more genomic VDR binding sites than untreated samples (treated: 2,776; control: 623) and overlap with enhancer histone markers (such as in *VDR* and *ALOX5*) resulting in the upregulation of the expression of 226 genes, and downregulation of three genes (116). Furthermore, after treatment with 1,25(OH)_2_D_3_ the cells showed a significant increase of intronic (treated: 36%; control: 26%) and intergenic (treated: 28%; control: 10%) binding sites (116). Interestingly, more than 40% of VDR binding regions overlap with strong enhancer regions in LCL, which is disproportionally more than that in non-immune cell types (117). Similarly, after 1,25(OH)_2_D_3_ treatment of THP-1 cells, a human monocytic leukemia cell line that resembles monocytes/macrophages, the VDR binding sites increased (treated: 1,820; control: 1,169) and shifted from the proximal transcription start site to distal regions (119). A comparison of multiple VDR ChIP-seq datasets from different cell types, including THP-1, LCLs (GM10855 and GM10861), LX2 (lung derived cell line), and LS180 (colon derived cell line), showed little overlap, suggesting a strong cell type specificity of VDR binding. For example, LPS-induced THP-1 cells, have 22% more VDR binding sites than that in the original THP-1 cells. Yet, they show more overlap of VDR binding regions compared with the overlap between monocyte and other different cell types (120). The overlap of VDR cistrome between two cell types with closer relationship is larger than overlap between cells with distant relationship: overlap between myeloid cells (monocyte/DC1/DC2) > overlap between myeloid cells and B cells > overlap between myeloid cells and stellate cells (53). However, despite these discrepancies between cell lines, the characteristics of the genomic changes after 1.25(OH)_2_D_3_ engagement of VDR imply a shift from an induction of gene expression through promoter engagement to a binding to potential enhancer regions both in LCL and THP-1 cells.

#### The Association of Nuclear VDR Binding and MS

By combining MS transcriptome data with VDR ChIP-seq data, the association between the VDR binding regions and characteristic MS genes has been studied. For example, after comprehensively analyzing vitamin D ChIP-seq data in LCL and whole blood mRNA transcriptome from MS patients and healthy controls, VDR binding sites were found to be enriched in genes that were differentially expressed in different forms of MS, such as primary progressive MS, secondary progressive MS, and relapsing–remitting MS, compared with that in controls (116). Furthermore, MS risk SNPs, such as rs703842, that are reported as eQTL in immune cells, are associated with transcription level of VDR bound *CYP27B1* (107).

Furthermore, genomic overlap and enrichment analysis pointed to disease risk SNP region that were enriched by VDR binding sites, and VDR binding sites with risk SNP located in. In CD4^+^ T cells, VDR-binding loci were enriched near SNP regions (100 kb around SNPs) that were associated with autoimmune diseases including MS and T-regulatory/T-helper cell functions (125). In LCL, VDR ChIP-seq data showed enrichment of VDR binding sites in GWAS identified intervals that were associated with multiple inflammatory diseases including MS, or *vice versa* autoimmune disease risk SNPs were found to overlap with VDR binding intervals (116). Furthermore, by supplementary annotation with epigenetic chromatin states data from ENCODE, both VDR-SE (strong enhancer) overlap and VDR-AP (active promoter) overlap are more likely to be present within MS risk regions comparing to the rest of the genome in LCLs (117). In myeloid cells, MS risk alleles, from three MS GWASs (17, 18, 133), are enriched (*p* < 10^−4^, not corrected for multiple testing) in VDR binding peaks (53).

### Potential Mechanisms of VDR Involvement in the Determination of MS-Associated Cell Fate

As mentioned earlier, the explanation for the effect of genomic regions with functional genomic annotation, such as annotating MS risk SNP in non-coding region with ENCODE data mentioned earlier, is an important addition to functional experimental studies. Functional experimental studies on causal SNP are mainly analyzing the potential interference between risk SNP alleles and gene expression or TF binding affinity using methods such as qPCR/microarray, Luciferase Reporter Assay or Electrophoretic Mobility Shift Assay. However, traditional experiments have some clear disadvantages, such as cumbersome, low-throughput methodology, hypothesis bias, or altered 3D structure of chromatin when using luciferase reporter. With the development of next-generation sequencing (NGS) and the dawn of the field of epigenetics, establishing annotation profiles has become easier. Especially, combined with NGS it was possible to define genomic SE structures and to identify their role in cell fate decisions, which made annotation more convincing and relevant. Below, we will discuss the definition of SEs and their association with the VDR and the role of VDR in dynamic chromatin landscape shaping processes.

#### Identification of Enhancer and SE Elements

Enhancers now can be identified by H3K4me1 (methylation of lysine 4 of histone 3), while active enhancers can be distinguished by H3K27ac (acetylation of lysine 27 of histone 3) and silent/poised enhancers by H3K27me3 (trimethylation of lysine 27 of histone 3) (134). Therefore, a genome-wide enhancer landscape of active enhancers was identified using the ChIP-seq method (135, 136). And as the association of enhancer with master TF (such as T-bet), pervasive coregulators (such as CREB-binding protein/p300, BRD4, and MED1) and DNA accessibility [such as regions identified by DNase-seq, formaldehyde-assisted isolation of regulatory elements-seq (FAIRE-seq) and Assay for transposase-accessible chromatin using sequencing (ATAC-seq)], they can also be used as an enhancer marker (31, 32, 137–139). Originally, the SNP loci associated with the greatest impact on cell fate or phenotype were assumed to be in the coding region of functional genes or in the promoter sequence of key TF genes (140, 141). However, with the impact of enhancers on cell identity and differentiation coming increasingly into focus, many functionally important SNP sites were found to be located in or in the vicinity of enhancers (142, 143). The recent observation that the regulation of lineage determining TFs, signal-dependent TFs, mediators, and chromatin-modifying coregulators were all governed by clusters of enhancers in a close spatial arrangement has led to the definition of SEs, which show higher enrichment for selected enhancer markers (31–33, 139, 144). Indeed, more risk SNPs have been found to be associated with SE structures of specific cell types than with normal enhancers (33, 143, 145). For example, type 2 diabetes-associated risk SNPs are located in enhancer clusters which are specifically active in pancreatic islets (146). Importantly, TFs can regulate cell-specific genes indirectly *via* SE region in other key TF genes, such as the *Bach2* gene in Th1/2/17 cells (32, 147). Different cytokine/cytokine receptor genes with SEs in different Th subsets (Th1/2/17) are all repressed by Bach2, whose gene also has an SE region in all these Th subsets, suggesting that SEs can shape a regulation network and that it will be possible to predict the role of TF genes in certain cell types (32, 147). Interestingly the *CYP27B1* haplotype is located in an SE region in mouse Th cells (dbSUPER database) (31). On the other hand, enhancers and SEs can themselves serve as transcription units and can generate non-coding and long non-coding enhancer RNAs. Their transcription is correlated with enhancer activities and also plays a regulatory role on the formation of enhancer–promoter loops and cell fate determination (112). Recently, one search identified a non-coding RNA, ThymoD, that was transcribed from the *Bcl11b* enhancer of an SE region. This RNA molecule can direct enhancer–promoter loop shaping, which further establishes T cell identity and blocks lymphoid malignancy (148). As only <20% of putatively causal SNPs affect TF binding, there needs to be another mechanism how risk SNPs exert their biological function. The functional failure of enhancer RNA caused by risk SNP allelic variations could be a possible explanation (112).

**Figure 1:**
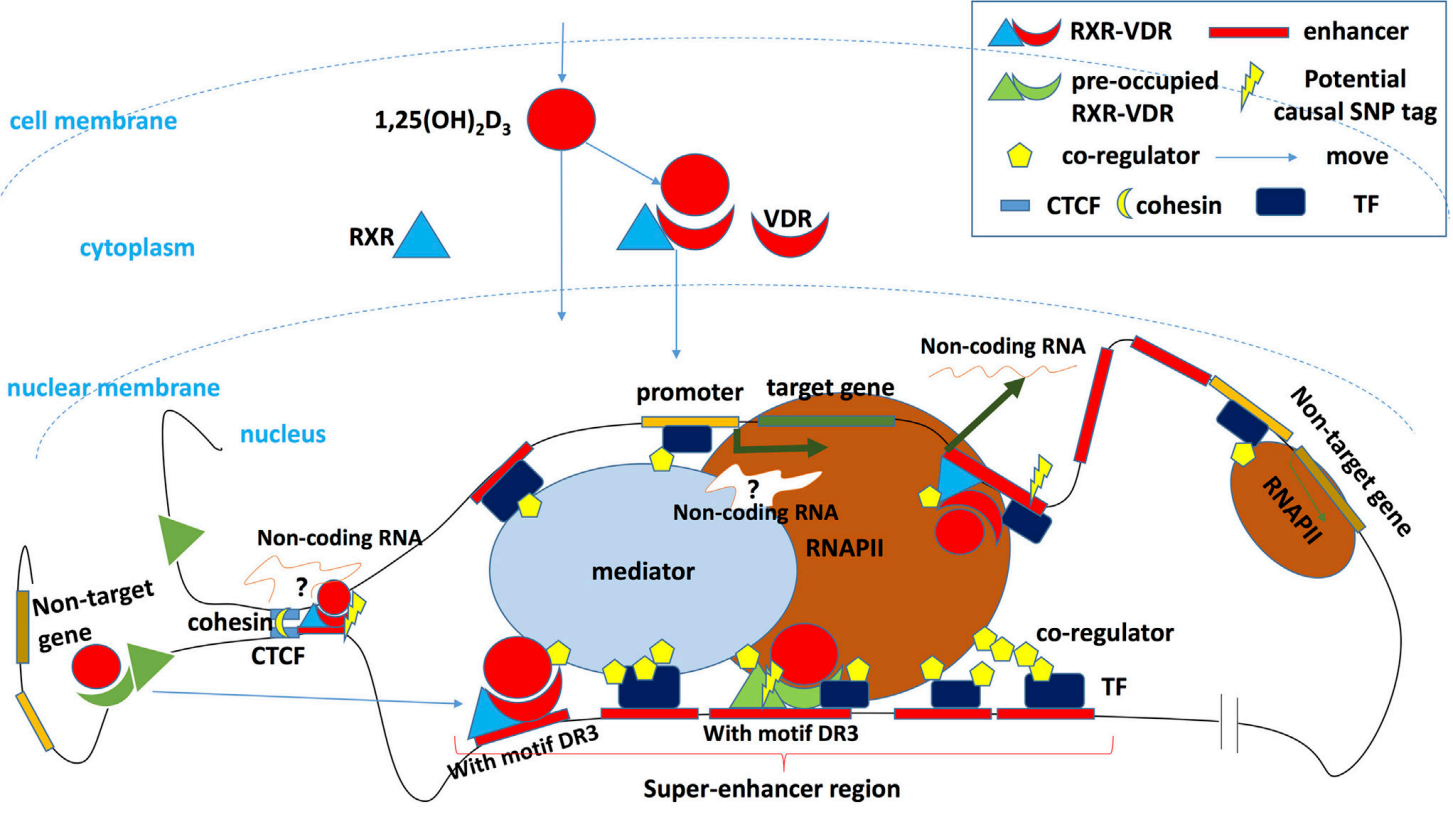
Vitamin D regulates the transcription of target genes. After binding its ligand 1,25(OH)_2_D_3_ and recruiting its partner retinoid X receptor (RXR), vitamin D receptor (VDR) enters the nucleus and binds to the transcription factor (TF) binding sites of target genes, where causal risk single-nucleotide polymorphism may be located. This induces transcription after the activation of other components of the transcription complex has been completed. A key enhancer can potentially be transcribed into a non-coding enhancer RNA, which regulates gene transcription or orchestrates cohesion-dependent looping to bring target gene promoter and enhancer region into a single-loop domain. The transcription activity of the whole domain is limited and regulated in a 3D loop isolated from other DNA regions by CCCTC-binding factor (CTCF) and cohesin (148), and some of CTCF overlap with binding of VDR (125, 126). Enhancers bound by VDR/RXR especially that in the so-called super-enhancers (SEs) or TF hotspot regions will recruit coregulators that shape chromosome landscapes and cooperate with other TFs. Other genes that are located in the 3D loop but outside the regulatory hub can also be transcribed but at lower rate. Outside the 3D loop, preoccupied VDR or RXR can be detected without vitamin D stimulation showing only low regulatory activity. Some preoccupied basal RXR binding sites can predict VDR binding sites following 1,25(OH)_2_D_3_ stimulation and could represent storage regions for the VDR in the absence of 1,25(OH)_2_D_3_. After ligand binding, these “stored” VDR can shift to a DR3 motif in intronic and intergenic regions of regulated genes (120). However, the preoccupied VDR region with DR3 motif plays a master (persistent/mother) enhancer role, persist, and will promote the whole region activation and SE shaping. Secondary VDR binding region without DR3 motif (suggesting it binds DNA *via* interaction with other pioneer TFs or *via* non-classic unknown RXR/VDR motifs) will constitute subservient (secondary/daughter) enhancers around master enhancers in SE region (121, 150).

Therefore, the fate of cells depends on chromatin modifications in cis-regulatory enhancer elements. The master TF are the major regulatory elements whereas SE regions and their modifications provide focal points on DNA sequences for those master TFs to act (149). After hormone or vitamin stimulation (such as estrogen or vitamin D) hormone ligand-induced SE regions are located around central enhancers with canonical hormone-specific regions (such as VDR DR3 elements or ERα dimer-specific elements). After ligand stimulation, subordinate enhancers would begin to shape the genomic environment around central enhancers together with motif enrichment of collaborating partners [such as Forkhead box protein A1 (FOXA1) or activator protein 1] (150). This is consistent with other research that shows that persistent VDR regions in THP-1 human monocytes show highly conserved binding of DR3-type motifs with high affinity after ligand stimulation (121). These persistent primary VDR loci in the same TAD will affect chromatin accessibility for further PU.1 and VDR binding, which leads to a general cell activation, while transient primary VDR loci regulate specifically their immune associated genes. As we mentioned before, SE, as a key regulatory region, is of great use for identifying key genes in cell fate determining. However, as SE defined by different enhancer markers will lead to different key regions, the relationship between SEs shaped by environmental signal TF and SE shaped by key TFs is still unclear. For example, in murine embryonic stem cells (mESCs), SEs shaped by key TFs (OCT4, SOX2, and NANOG) are correlated with and provide a platform for signal TFs (STAT3, SMAD3, and TCF3), but in MCF-7 cells, ERα, and FOXA1-shaped distinct SE regions. Therefore, focusing on the effect of the modification of regulatory hubs in the presence of VDR can help our understanding of vitamin D’s effect on specific cell types (Figure 1), and the clarification of relationship between VDR and SEs in multiple immune cell types would further the understanding of MS pathogenesis.

#### The Hierarchical and Chronological Order of VDR and Other TFs Binding to Key Enhancers during Cell Differentiation

Recent immune cell plasticity research emphasized the importance of TFs that are dependent on environmental signals, such as VDR (vitamin D-dependent), SMAD3 (TGF-β-dependent), and STAT (cytokine dependent), which participate in the modification of enhancer landscapes and interact with master TFs, such as T-BET, PU.1 and OCT4. In macrophages and B cells, the master TF PU.1 plays a pivotal role in shaping a cell-specific epigenetic genomic landscape to enable the subsequent binding of environmental signal-dependent TFs that directly regulate gene expression (151). In mESCs, myotubes and pro-B cells, cell type-specific master TFs such as OCT4, MYOD1, and PU.1 also modify the chromosome landscapes and direct TGF-β/SMAD3 to bind different identity gene loci separately (152). Indeed, master TF-deficient cells show only limited impact on establishing the mature phenotype in CD4^+^ T cells, and the pivotal factors that shape epigenetic genomic landscapes in T cells are still elusive. However, there are some clues. Signal-dependent TF, such as STATs, have an important role in chromosome modification during T cell differentiation. For example, STAT4 promotes H3K4me3 modification on Th1 cell identity genes, and STAT6 modifies H3K27me3 in gene loci of Th2 cell identity genes (153). While the expression of master TF (e.g., T-bet) in signal-dependent TF-deficient cells (e.g., STAT4-deficient cells) fails to recover enhancer landscape changes and consequently, the cell phenotype (e.g., Th1 cells) (123, 147, 152, 154, 155). In Th17 cells, BATF and IRF4, not Th17 master TF RORγt, cooperate to modify chromatin landscapes including genome-wide distribution of p300 allowing other factors such as STAT3 and RORγt to attach (123). In general, it becomes clear that an underlying epigenetic instability is an important principle in the cell fate determination and the observed plasticity of cell subsets (156). Environmental signal-dependent TF potentially play an equal role with master TF, which of them is the pioneer factor that opens the chromatin is still uncertain. Therefore, the role of VDR in cell fate determination although not clearly elucidated could be larger than expected.

It is noteworthy that TF binding is always dynamic and transient rather than stable which makes the molecular basis of differentiation even more complex. In some cases VDR can bind to positions before actual 1,25(OH)_2_D_3_ stimulation. After stimulation, the VDR binding profile will shift to new positions (119). Finally, multiple factors can bind certain DNA regions in turn according to the dynamically changing epigenetic landscapes shaped by binding TFs. For instance, cyclical chromatin looping and TF binding of the regulatory region of p21 gene has been observed after stimulation with 1,25(OH)_2_D_3_ (157).

## Conclusion and Perspectives

Overall, the role of the VDR cistrome in the epigenomic shaping of the enhancer landscapes and cell type determination will affect the maturation and differentiation of immune cells that are involved in the pathogenesis of MS. We suggest that this may be a key to the explanation of the biological contribution of risk-associated vitamin D SNPs, as well as environmental levels of vitamin D and MS risk.

In the future, we will benefit significantly from new technologies. High throughput methods, such as RNA-seq, ChIP-seq, updated ChIP-seq with higher resolution (ChIP-exo), chromatin interaction analysis by paired-end tag sequencing (ChIA-PET), high-resolution chromosome conformation capture (Hi-C) are providing a more complete and unbiased view of the TF cistrome and TF networks. Taken together with traditional experiments, high throughput methods and bioinformatics, great advancements have been made in analyzing the regulation, interactions and functions of VDR and its ligands in immune cells. Further exploration will help to understand this highly dynamic and environmentally responsive network and answer the question how individual variations in environmental levels of vitamin D, genotypes, and risk alleles, in particular, influence or modulate outcomes in immune cell differentiation and predispose individuals to autoimmune diseases such as MS. MS epidemiology researchers can design clinical studies that include new data from NGS *via* increased coordination and collaboration with genomics/epigenomics labs, which will potentially connect changes on MS risk genomic regions to clinical processes directly and lead to new insights in MS pathogenesis and new diagnosis/treatment targets.

## Author Contributions

ML wrote and edited the manuscript, BT and HK edited the manuscript.

## Conflict of Interest Statement

The authors declare that the research was conducted in the absence of any commercial or financial relationships that could be construed as a potential conflict of interest.

## Abbreviations

AP-1, activator protein 1; ATAC-seq, assay for transposase-accessible chromatin using sequencing; C/EBP, CCAAT/enhancer-binding protein; ChIA-PET, chromatin interaction analysis by paired-end tag sequencing; ChIP-seq, chromatin immunoprecipitation sequencing; CIS, clinically isolated syndrome; CNS, neurodegenerative central nervous system; CREB, cAMP response element-binding factor; CSF, cerebrospinal fluid; CTCF, CCCTC-binding factor; DC, dendritic cell; DHS, DNase hypersensitivity sites; DR3, a direct repeat of 2 hexameric core binding motifs with a spacing by 3 nucleotides; EAE, experimental autoimmune encephalomyelitis; EMSA, Electrophoretic Mobility Shift Assay; eQTL, expression quantitative trait loci; FAIRE-seq, formaldehyde-assisted isolation of regulatory elements-seq; FOXA1, Forkhead box protein A1; GABPA, DNA-binding subunit of GABP; GR, glucocorticoid receptor; GWAS, genome-wide association studies; Hi-C, high-resolution chromosome conformation capture; LCL, lymphoblastoid cell line; LD, linkage disequilibrium; MBP, myelin basic protein; MED1, mediator complex subunit 1; mESC, murine embryonic stem cell; MRI, magnetic resonance imaging; MS, multiple sclerosis; NcoR1/2, nuclear receptor co-repressor 1/2; NGS, next-generation sequencing; NK, natural killer; PLP, proteolipid protein; PTH, parathyroid hormone; RRMS, relapse-remitting multiple sclerosis; RUNX2, Runt-related transcription factor 2; RXR, retinoid X receptor; SE, super-enhancer; SMRT, silencing mediator of retinoid and thyroid hormone receptor; SNP, single-nucleotide polymorphism; SRC1, steroid receptor coactivator 1; TAD, topologically associated domains; TF, transcription factor; VDR, vitamin D receptor; VDRE, vitamin D responsive elements.
